# Assessing Current and Future Freshwater Flood Risk from North Atlantic Tropical Cyclones via Insurance Claims

**DOI:** 10.1038/srep41609

**Published:** 2017-02-02

**Authors:** Jeffrey Czajkowski, Gabriele Villarini, Marilyn Montgomery, Erwann Michel-Kerjan, Radoslaw Goska

**Affiliations:** 1 The Wharton School, University of Pennsylvania, 3730 Walnut Street, Philadelphia, PA 19104, USA; 2 Willis Research Network, 51 Lime Street, London EC3M 7DQ, UK; 3 IIHR-Hydroscience & Engineering, The University of Iowa, 100 C Maxwell Stanley Hydraulics Laboratory, Iowa City, IA 52242, USA

## Abstract

The most recent decades have witnessed record breaking losses associated with U.S. landfalling tropical cyclones (TCs). Flood-related damages represent a large portion of these losses, and although storm surge is typically the main focus in the media and of warnings, much of the TC flood losses are instead freshwater-driven, often extending far inland from the landfall locations. Despite this actuality, knowledge of TC freshwater flood risk is still limited. Here we provide for the first time a comprehensive assessment of the TC freshwater flood risk from the full set of all significant flood events associated with U.S. landfalling TCs from 2001 to 2014. We find that the areas impacted by freshwater flooding are nearly equally divided between coastal and inland areas. We determine the statistical relationship between physical hazard and residential economic impact at a community level for the entire country. These results allow us to assess the potential future changes in TC freshwater flood risk due to changing climate pattern and urbanization in a more heavily populated U.S. Findings have important implications for flood risk management, insurance and resilience.

During the first decade of the 21^st^ century, there has been an increase in North Atlantic TC activity, with most of the ten costliest U.S. TCs of all time also occurring in this timeframe including Katrina, Sandy and Ike^1^. While wind-related damages were certainly substantial for these events, massive TC flood damages also ensued. TC flood damages can either be storm-surge or freshwater (due to heavy rain, which itself can occur in both coastal areas and inland) driven. However, media coverage and warnings have tended to focus on potential wind losses, and more recently, also on storm surge stemming from Hurricanes Katrina and Sandy. This might be somewhat shortsighted since freshwater flooding from TCs has been shown to have substantial socio-economic impacts, killing hundreds and causing billions of dollars in damage^2^,^3^,^4^,^5^,^6^, affecting large U.S. geographic areas that are not limited to the coast^7^,^8^, and potentially having the most severe effects hundreds of kilometers away from the center of circulation^5^,^7^,^9^,^10^.

Yet, despite this growing scientific recognition, a comprehensive assessment of the freshwater flood risk – linking physical hazard to economic impacts at a community level over the entire path of the storm– has yet to be realized, with some preliminary efforts only geared towards specific events^5^. Here we systematically assess freshwater flood risk through an interdisciplinary approach that analyzes the full set of all 28 significant U.S. landfalling TC related flood events (https://www.fema.gov/significant-flood-events) having occurred from 2001 to 2014 (Supplementary Table 1 and Supplementary Figure 1).

We show that, on average, each TC affected nearly 8,000 communities across the country. Communities do vary markedly by land size and population, but on average this translates into over 40 million total housing units and 93 million people along the entire TC path. Across the 28 TCs, and focusing only on flood not wind, we find that about one third of the total residential flood insurance claims were related to storm surge, with the remaining two thirds thus being freshwater driven‒and these freshwater specific claims being almost equally located between coastal communities (55 percent) and inland areas (45 percent). While this confirms TC coastal flooding risk, we also demonstrate the significant spatial extent of TC freshwater flooding for inland communities.

The number of claims from the federally-managed National Flood Insurance Program (NFIP) – the main provider of flood insurance in the U.S. - serves as the basis for our statistical modeling to identify the major drivers associated with freshwater flood losses. We then use our derived statistical relationships to understand future flood risk given potential increases in flood hazard due to a warmer climate and expanding urbanization. We find that there could be up to a 17% increase in residential losses (again, using the number of NFIP insurance claims as a proxy) for a 20% increase in flood magnitude, and a 2.4% increase due to changes in impervious surface of the same magnitude as what experienced over the first decade of the 21^st^ century. Since losses from major TCs can be in billions of dollars, this constitutes a significant exposure increase for the communities at risk. Thus our findings provide the details to comprehensively assess TC freshwater flood risk now and under plausible changing conditions both in terms of flooding and urbanization. In a world of increasing natural disaster risk, this view represents an important move toward greater natural disaster resilience.

## TC Freshwater Flood Hazard Assessment in U.S. Communities

Following from a quantitative risk modeling framework we first assess and then statistically model the relationship among TC freshwater flood hazards, socio-economic exposure and economic impacts focusing on residential losses (see Methods section for an overview of this process). From a hazard perspective, we leverage daily discharge data from 3,035 U.S. Geological Survey (USGS) stream gages to assess the TC freshwater flood hazard at a community level.

The US Federal Emergency Management Agency (FEMA) defines a community as a political entity (e.g., city, town, county) having floodplain ordinance authority^11^, thus a community serves as our primary unit of analysis. Across all 28 TCs a total of 218,146 communities were affected in 38 of the 50 U.S. states. On average 7,791 communities were affected per event, with Ike (2008) affecting the most (16,060 communities) and Wilma (2005) the least (617 communities). The maximum number of times an individual community was affected was 21, or by 75% of the TC events considered here, with affected communities extending well inland (Supplementary Figure 2). Although we find that the vast majority of these affected communities (90%) experience a TC freshwater flood hazard below flood stage level (defined in Methods section), this expansive geographic extent of the flood hazard begins to highlight the broad scope of potential flood risk.

**Figure 1:**
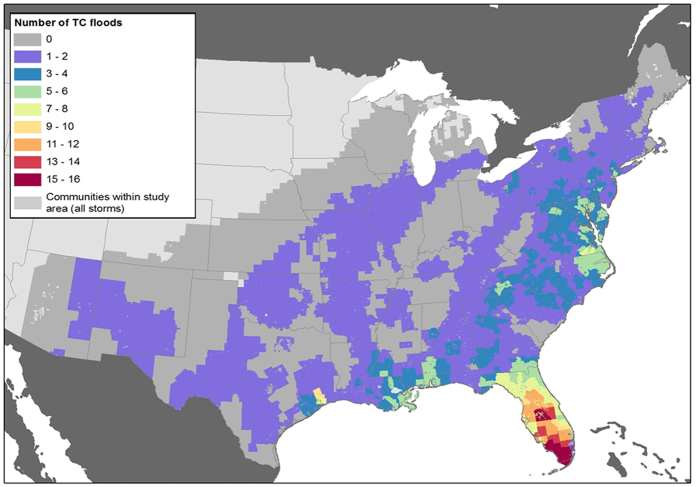
Flooding associated with TCs. This map shows the number of flood events (flood ratios larger than 1) in each of the FEMA defined communities that have been within 500 km of the passage of any of the 28 significant flood event TCs considered in this study (2001–2014). Maps were created in ESRI ArcGIS version 10.2.2 (http://www.esri.com/software/arcgis/arcgis-for-desktop).

The 28 TCs in this study have indeed been responsible for flooding and major flooding (see Methods) over large areas of the central and eastern U.S. affecting a total of 21,705 communities, or approximately 775 communities on average per event (Fig. 1). As expected, the Gulf Coast and the U.S. eastern seaboard (and Florida in particular) are the areas that have been affected the most by TC flooding, representing 55% of the total 21,705 communities affected by both flooding and major flooding over the period 2001–2014. However, the extent of the freshwater flood hazard is not limited to these coastal areas, with the remaining 45% of the communities affected by flood and major flood effects over that same period being in inland, i.e., non-coastal, states such as Illinois, Missouri, Pennsylvania, Ohio, and West Virginia. And while nine coastal states - Florida, Texas, Louisiana, New Jersey, South Carolina, New York, North Carolina, Virginia, and Georgia - represent nearly 75% of the total 5.6 million flood insurance policies covered by the NFIP^12^, we still found that about 200,000 policies on average per event are in-force in inland states, a non-trivial amount at risk. (Supplementary Figure 3). Lastly, and as was found with Hurricane Ivan^5^, the freshwater flood TC hazard is not limited to communities that participate in FEMA’s flood program, highlighting flood hazards (Fig. 1) that occur in areas with no corresponding flood insurance protection in place (Supplementary Figure 3), and thus likely with limited overall floodplain management.

## Using Flood Insurance Claims as a Loss Proxy

While we have provided an assessment of the freshwater flood hazard across the entire extent of the TC affected areas, a comprehensive view of the TC freshwater flood risk is the combination of both the flood hazard and its associated economic impacts. We access data from the NFIP and catalog residential freshwater flood insurance claims associated with these 28 TCs as our measure of impacts in affected communities. While flood insurance markets vary across countries, in the U.S. this federal insurance program is the primary residential insurer of flood risk and we draw upon its extensive claim database with over 2 million records. Subsequently though, since the NFIP claim portfolio only encompasses governmentally insured residential losses the loss data should be considered a lower bound estimate of the total freshwater flood economic impacts experienced in all flood hazard affected areas shown in Fig. 1. In terms of the freshwater flood impacts from these 28 TC events, we catalog a total of 443,484 residential freshwater flood insurance claims incurred, or an average of 15,839 per event. While we focus on the number of claims incurred, we can take the $34,000 mean claim values from a recent study that analyzes 35 years of operation of the NFIP^13^ to estimate corresponding average residential damage amounts from these incurred claims at over $530 million per TC event.

**Figure 2:**
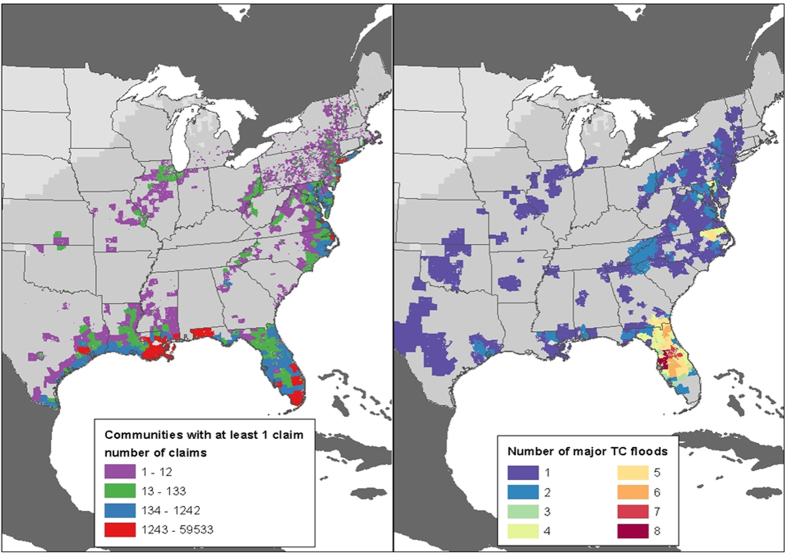
Communities with at least 1 residential claim (left panel) and communities impacted by at least one major TC flood over the period 2001–2014 (as defined by flood ratio over 2.2, corresponding to major flooding; see Methods) (right panel). Maps were created in ESRI ArcGIS version 10.2.2 (http://www.esri.com/software/arcgis/arcgis-for-desktop).

Figure 2 (left panel) illustrates the location of all the communities with freshwater flood claims and Fig. 2 (right panel) shows the numbers of times communities have experienced a major TC flood (defined as having a flood ratio value over 2.2; see Methods and Supplementary Figure 4). As expected, the locations of TC flood hazards and the majority of flood insurance policies-in-force cause claims to be highly concentrated in coastal communities. But again, freshwater flood losses are not limited to these areas with significant claim amounts in inland areas of the U.S., including Illinois, Missouri, Pennsylvania, Ohio, and West Virginia. Finally, FEMA designates special flood hazard areas (SFHA) as being high-risk. Still, we find that 26% of the total 443,484 claims incurred by the 28 TC freshwater flooding events we studied were actually outside of those areas, where the risk is advertised to residents by the federal government as being low. This low-risk area result is consistent with other recent NFIP claim findings^13^.

## Modeling Community Freshwater Flood Hazard to Flood Losses

Currently, the National Weather Service (NWS) only stipulates that areas exposed to a flood hazard are at simply an increased threat to property, something we would like to better quantify for risk assessment. Thus, we move to the statistical modeling of the number of NFIP insurance claims incurred at each NFIP community in terms of the key drivers of this impact reflecting not only the flood hazard, but also relevant exposure and vulnerability components of that community (see the Methods for a description of the modeling framework we adopt). The freshwater flood hazard component is represented by the TC discharge values normalized by the at-site two-year flood event, and categorized as bankfull, minor, moderate and major flooding (we refer to this as the flood ratio; see Methods). To quantify exposure and vulnerability, we use variables related to the community’s distance from the coastline, whether it is located within a coastal state, its percentage of impervious surface (to examine the effects of urbanization and based upon 2006 values), the proportion of the community in low, medium or high risk areas, the number of housing units (based on the 2010 values), and the number of FEMA residential flood insurance policies-in-force in the community per year of the event. For statistical power purposes we pool the data from all the 28 TCs; but as these are different types of TC flood events we also control for any unobserved event-specific fixed effects through event dummy variables represented by the TC intensity at landfall (Supplementary Table 1). We also include year variables to control for any unobserved time-specific fixed effects.

Table 1 presents the results from our empirical analysis where we model the count of claims for all 150,546 communities with at least one residential insurance policy-in-force, and further utilize a zero-inflated negative binomial regression (ZINB) model with robust standard errors to account for the large number of these communities with zero claims incurred (see Methods section for a description of the statistical analyses employed). The Wald test of the joint insignificance of our explanatory variables is rejected at the 1% level, with 20 of the 24 individual predictors in the model significant at the 10% level or less, including notably the flood hazard variables at the 1% level. Based on our modeling results (Table 1), flood conditions lead to an increase in the number of insurance claims, when compared to bankfull conditions. We find a non-linear marginal increase for higher flood magnitude when compared to the bankfull conditions: the expected number of claims increases by a factor of 2.5 for minor flooding, 4.9 for moderate, and 12.1 for major flooding. The impact of flood hazard risk is also further quantified by examining the inherent proportion of each community in low-, medium- or high-risk areas as designated by a 1996 Natural Disaster Study conducted by the FEMA on behalf of the U.S. Department of Transportation Pipeline and Hazardous Materials Safety Administration (see Methods section for further overview). For each community we calculated the proportion of area representing three levels of low, medium, and high flood risk. These results indicate that there is a larger number of claims in those communities that have a higher proportion of high flood risk as would be expected.

The number of insurance claims resulting from a tropical storm compared to a Cat 1–2 hurricane are smaller by a factor 0.1, while the claims associated with major hurricanes (Cat 3–5) increase by a factor of 9.9 on average. A possible explanation for these results is tied to the larger size and rainfall amounts in hurricanes compared to tropical depressions and storms^14^, potentially leading to saturated ground and higher flood magnitudes. As shown in Supplementary Table 1, the median radius of the outer closed isobar at landfall is 175 nm for tropical storms, 232.5 nm for Cat 1–2 hurricanes, and 300 nm for Cat 3–5 hurricanes.

Whether the community is located along the coast or further away from it also plays a role where we estimate a reduction in insurance claims by a factor of 0.6, 0.6, and 0.2 for communities located in distance bands 25 to 100 miles, 100 to 500 miles, and greater than 500 miles from the coast, respectively. Finally, we also find that there is a 100% increase in the average number of expected insurance claims per community for a 1% increase in impervious surface. From a hydrologic standpoint, we expect larger flood peaks in urban rather than rural settings because of the limited infiltration, leading to faster response and larger peaks. These results indicate that urbanization and conversion of the landscape towards impervious surface has played a significant role in increasing the impacts of TC flooding. As expected, the count of claims increases as the number of residential policies in-force increases, but decreases as housing units increase. This accounts for relatively low market penetration rates, especially outside of the FEMA-defined high risk special flood hazard areas (SFHAs). Finally, the two zero-claim probability explanatory variables in the inflate portion of the model (i.e., maximum flood ratio observed and the log of residential policies in force) have the expected sign (i.e., higher values of each lead to lower probability of observing 0 claims incurred) and are statistically significant at less than the 1% level.

To test the robustness of our results we have also estimated additional model formulations summarized in Supplementary Table 2 (models A to C) where the dependent variable now is SFHA, Non-SFHA, and Single Family residential only number of claims, respectively. We see that the overarching message does not change from the results detailed in Table 1. However, one interesting change here is that for an impacted state the number of SFHA claims is lower in a coastal state compared to non-coastal states, whereas the number of Non-SFHA claims is higher in a coastal state compared to non-coastal states, everything else being equal. This again highlights the extent of TC freshwater flood risk well inland, as well as the extent of freshwater flood risk in coastal areas not limited to FEMA designated high risk flood areas.

**Figure 3:**
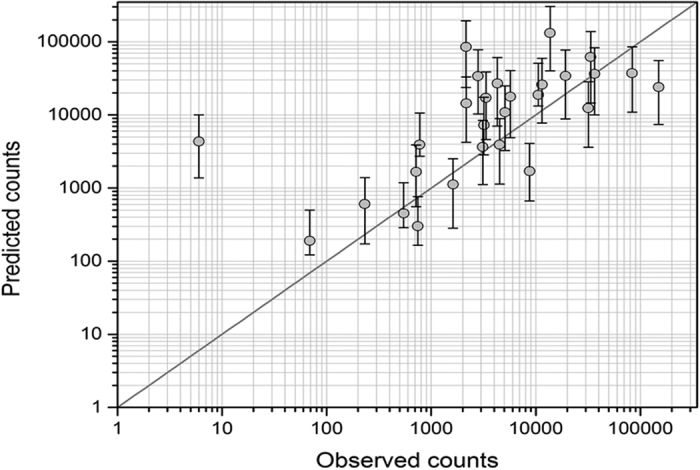
Fit of Predicted vs. Actual Claims Incurred per TC. Log scales of actual and predicted claims are taken and 95% confidence intervals on the Table 1 estimated coefficients are shown for predicted counts. Each observation represents one of the 28 TCs.

We validate our regression results in two main ways: 1) using the results from Table 1 (including the confidence intervals on our estimated coefficients) we compare the fit of predicted vs. actual flood claims per each of the 28 TCs (Fig. 3); and 2) we apply a k-fold cross validation on the model in Table 1 to evaluate its ability to fit out-of-sample data (see the Methods section for a description of the cross-validation statistical analyses employed). Figure 3 shows that for 17 of the 28 TC events the actual number of claims incurred falls within the 95% confidence bound of predicted counts estimated from our model, providing further validity to our estimated model. The pseudo-R-squared statistics generated from k-fold cross validation (Supplementary Table 3) indicate that the ZINB models consistently capture about 12% of the variation in out-of-sample test data, with a high value of 52%. The cross-validation goodness-of-fit results are lower than the full model results with pseudo R-squared values here closer to 30%. Importantly though, in all k-fold estimation, the key flood ratio variables of interest perform as they did in Table 1 in terms of magnitude, sign, and statistical significance, providing further validation of our findings.

## Future Changes in TC Freshwater Flood Losses

These results have unveiled and further quantified some of the major drivers responsible for the observed residential freshwater flood loss from U.S. landfalling TCs including the flood hazard magnitude and the level of urbanization (impervious surface coverage). Different modeling studies point to an increase in U.S. TC rainfall up to ~20% in a warmer climate^15^,^16^,^17^,^18^. Moreover, large areas of the continental U.S. that are under threat from TC strikes have also experienced increasing urbanization.

Significantly our methodology allows us to predict via a comparative statics analysis how changes in the climate system and urbanization might impact residential loss, assuming that all the other exposure and vulnerability factors stay the same. As an illustration, we select two recent TC events, Hurricanes Ike (2008) and Irene (2011), and determine what the local and regional residential economic impacts of those two disasters would likely be if flood magnitudes were increased up to 20% under the present level of exposure and vulnerability. Similarly, we measure what the effects would be if urbanized areas increased under the present level of flood hazard. We examine these two hurricanes as representative of high impact storms affecting the eastern and central U.S.

Table 2 illustrates that by increasing the observed Hurricanes Ike and Irene TC flood ratios by 1%, 5%, 10%, and 20% at every USGS stream gage location (Supplementary Figure 5 illustrating Hurricane Ike only), we obtain increased predicted loss amounts of 0.7%, 3.5%, 8.6%, and 17.1%, respectively (measured by expected flood insurance claims). These increases are compared to predictions of greater urbanization from increases in impervious surfaces based on the 2001 to 2011 percentage increases, and assuming this rate of change to persist to 2021. Predicted claims increase by 2.4% with the increased urbanized land-use. In other words, increasing urbanization at its continued recent pace is roughly equivalent to a 3% increase in TC flooding.

## Conclusion

This novel study characterizes U.S. communities that have been at large risk of freshwater flooding from all substantial TCs between 2001 and 2014. We show that the number of residential losses from that type of flooding were actually twice as high compared to storm surge losses (using the number of residential flood insurance claims of the U.S. federal flood insurance program as a proxy). Furthermore, freshwater flood impacted areas from coastal versus inland freshwater flooding were divided 55/45 percent. We are also able to determine the relationships between intensity of the TC flood event (measured by the flood ratio) and losses, under current and future conditions. Thus our results can provide a new way to undertake flood risk assessment across all areas potentially impacted by TCs, not just coastal landfall locations.

Significantly though this work has important flood risk management implications, particularly in the U.S. Generally, flood risk management surrounding an event occurrence can either be ex-ante or ex-post, with ex-ante activities focused on risk awareness, measurement, and risk-based decision making, while ex-post activities are centered on event response and recovery. Often in the U.S. flood risk management is primarily ex-post response driven by media attention focused on the coastal landfall impacts that are most visual and concentrated. But our findings shed new light on the overall damage triggered by those TCs, along their entire path inland, not just on the coast.

In a recent report^19^, the U.S. National Weather Services (under NOAA) which provides the official TC alert for the nation, states that improvement in how it communicates and educates on the risk of inland flooding was the number one overreaching recommendation. Hurricane Matthew, which skirted up the U.S. Southeastern Atlantic coast in October 2016 eventually making landfall in South Carolina as a category 1 hurricane, is a recent and poignant reminder of the U.S. TC inland flood threat. Initial estimates of inland flood losses represented about half of the insured losses and three quarters of total loss (insured and uninsured) including both wind and surge loss estimates^20^. And its over 19,600 NFIP claims filed to date^21^ would make it the 8^th^ largest number of flood claims incurred amongst our 28 storms analyzed here. Highlighting the areas susceptible to extensive inland flood losses from TCs – as we have done here – is a critical step to better community preparation toward future events from an ex-ante risk management awareness perspective.

Moreover, FEMA has also recently been tasked with remapping areas across the country for flood risk and a number of U.S. inland flood catastrophe models have been/are in development in the private sector. The NFIP is slated to be reauthorized and possibly modified by September 2017. Key policy debates around the NFIP reauthorization will likely focus on the role of the private insurance market and the use of the more sophisticated catastrophe modeling to allow for more accurate maps and risk-based pricing as well as expanded insurance coverage, especially in non-coastal parts of the U.S. Our work here comprehensively assesses TC freshwater flood risk in all areas now and under future conditions, and should therefore be useful to these endeavors by providing an additional level of insight that is valuable for better price measurement and understanding of the flood risk across all sources of flood hazard. Something that has been called for within the risk management industry is to better assess catastrophe risk^22^,^23^,^24^.

As we close a few caveats should be mentioned. From an impact perspective, losses here are limited to insurance claims from insured properties in participating NFIP communities. While our NFIP dataset is robust in this regard as the primary residential property insurer for flood in the U.S., uninsured losses are not accounted for in our TC flood risk assessment and thus the true flood loss footprint is not complete. Similarly, we are only modeling the number of claims incurred, not the associated property dollar loss from each claim which would not be equivalent for all claims. In order to better assess this, our flood ratio approach would need to be enhanced to account for flood severity (i.e., inundation depths) per stream gauge location, and then additional exposure controls accounting for structural and elevation characteristics would need to be incorporated into the statistical analysis. Also, we focused on residential loss, which is only a portion of the total losses (e.g. business, public infrastructure, interruption of economic activities). From a flood hazard perspective, we have interpolated flood hazard data to the communities using inverse distance weighting, and it is possible that other interpolation methods could have provided different results. The stream gage network we used is very dense, however, mitigating the potential issues associated with the selected interpolation scheme.

## Methods and Associated Data

For our study quantitative risk modeling of TC freshwater flood risk is ultimately deriving the statistical relationship among TC freshwater flood hazards, socio-economic exposure and economic impacts from the set of all 28 significant U.S. landfalling TC related flood events having occurred from 2001 to 2014. In order to statistically model the TC freshwater flood risk we must first provide in all affected communities an assessment of the flood hazard, an assessment of the economic impacts (i.e., losses), and also control for any socio-economic exposure variation. Therefore, our overall methodology to comprehensively assess the TC flood risk involves three main components: 1) provide an assessment of the flood hazard; 2) provide an assessment of the economic impacts; and 3) combine these two components in a statistical estimation to determine the relationship between hazard and loss that also controls for relevant socio-economic exposure characteristics of communities that may also contribute to loss results. We provide details on each of these phases below:

### TC Flood Hazard Assessment

The best TC track data available from the Hurricane Database (HURDAT database; ref. 25), maintained by the U.S. National Hurricane Center (NHC), provides information about the recorded TC position and intensity. These data include latitude and longitude of the center of circulation of the recorded storms, maximum sustained wind and minimum central pressure every six hours during the storm lifetime. USGS stream gage daily discharge measurements are used to: 1) compute the 2-year flood peak discharge, which we use as normalizing factor to perform analyses at the regional scale; 2) to identify peaks associated with TCs. The selection of the 2-year return period is due to two main reasons: first, it is roughly the discharge value corresponding to bankfull conditions, with values larger than it pointing to out-of-bank flow (Supplementary Figure 4); second, it can be estimated accurately from the data using a 30-year window. Because of the large human modifications of most of the watersheds over the study region (changes in land use/land cover, construction of dams; refs 26 and 27), we compute the 2-year flood peak at a given location using annual maximum peak daily discharge over the 1980–2013 period. Here we only consider USGS stream gages with a record of at least 20 complete years (a complete year is defined as having at least 330 daily values) over the 1980–2013 period. We define flooding associated with a TC as the largest daily discharge value measured by a station located within 500 km from the center of the storm during a time window of two days prior and seven days after the passage of the storm^8^,^28^,^29^,^30^. Within our domain, there are 3,035 stream gages that satisfy these conditions (Supplementary Figure 1).

One of the possible limitations in dealing with streamflow data at the regional scale is that there is an intrinsic dependence of discharge on drainage area that needs to be accounted for refs 7, 31 and 32. One approach to address this is to combine observed high-resolution rainfall fields with hydrologic and hydraulic models to reproduce observed discharge records. While potentially interesting, the development of hydrologic models over large areas is subject to a number of obstacles^33^, including changes in land use land cover and discharge regulation by dams and reservoirs. Because of the high density of USGS stations over the study region (Supplementary Figure 1), we follow a data-driven approach and normalize the peaks caused by TCs by the at-site 2-year flood peak. The ratio that we obtain by normalizing the TC flood peak and the 2-year flood event will indicate how much larger than the 2-year flood event the flood caused by TCs was, accounting directly for dependencies on drainage area. A flood ratio of 1 indicates that the storm caused a flood peak of the same magnitude as the 2-year flood event. Flood ratio values larger (smaller) than 1 point to flooding larger (smaller) than the 2-year event.

Using the flood ratio values estimated at each of the 3,035 USGS stream gage for each TC in our study, we interpolated flood hazard data to the communities. A 100-m resolution raster grid was created for each of the 28 TCs using the inverse distance weighting (IDW) spatial interpolation method in ArcGIS and the point locations of each USGS stream gage located within 500-km of the TC tracks in our study area. The IDW interpolation was controlled by setting the minimum and maximum number of neighbor observations at 3 and 5 respectively. For data points that are not uniformly distributed, defining the minimum and maximum number of neighbor observations for interpolation is a better suited approach than using a certain distance bandwidth to define neighborhoods. The maximum value of the flood ratios interpolated by the IDW grid within each community was assigned as the value for the entire community. Although this is a rough estimation, an advantage of the IDW method is that it does not estimate values that are higher than the observed input data unlike some other spatial interpolation methods. The maximum values from the interpolated grids, as well as all other predictor variables, were estimated for each community.

One of the potential limitations of using the flood ratio is that it does not generally provide information regarding the severity of the flood event. To address this issue and similar to ref. 8, we use the flood status classification by the National Weather Service (NWS), which generally defines the flood level into five flood categories: Action, Bankfull, and Flood Stage (above which there is a hazard to lives, property and commerce), which is further divided into Minor, Moderate and Major Flooding.

For each of these categories, we have computed the corresponding value of flood ratio for all the USGS stream gages within our region of interest for which the NWS has created this classification. Based on the results in Supplementary Figure 4, we consider flood ratio values smaller than 1 as bankfull conditions (i.e., the water is still within the river banks), while values of 1 and larger are indicative of flood conditions. We further stratify flooding into *minor* (flood ratios between 1 and 1.5), *moderate* (flood ratios between 1.5 and 2.2) and *major* (flood ratios larger than 2.2).

### Flood Loss Assessment

The information about residential flood insurance claims associated with floods from TCs is based on the data from the US Federal Emergency Management Agency (FEMA) which manages the national flood insurance program (NFIP). Since 1968 flood insurance has been available in the U.S. through this federal government program because private insurers contended at the time (and still primarily do today) that the peril was uninsurable by the private sector for a number of reasons we will not elucidate on here (see ref. 34 for a review). Thus, this FEMA insurance program represents the main source of data for flood claims and damage for any state in which an active NFIP community is located.

This national program, which does not cover any other hazard but flood, has grown substantially over its 48 years of operation and provides insurance to 5.1 million policyholders across the country today in exchange for $3.5 billion in premiums paid as of December 2015. The program now covers more than $1.2 trillion in assets, a 250% increase since 1990 (corrected for inflation)^34^,^35^. We have access to all flood insurance policies in a particular year over the 2000–2012 period, and we have access to the insurance claims database over this same time period with claim loss information on individual policies for the entire country. “Residential” equates to single-family, two- to four-family, and other residential structures. Non-residential (i.e., primarily commercial) structures covered by the NFIP, less than 5% of the total insured portfolio, are excluded from this analysis. Because we are focused on analyzing freshwater flood losses, we exclude all claims explicitly due to “tidal water overflow” as classified by the FEMA (i.e., storm surge losses). The FEMA insurance portfolio does not contain individual residential location (street address), therefore we have aggregated the data to the community level (as designed by FEMA). The geospatial dataset for communities is derived from the 2010 community layer geodatabase (https://data.femadata.com/ last accessed 4 February 2016).

### Statistical Modeling and Validation

The dependent variable in our multivariate analysis is the number of residential flood insurance claims occurring during any of the 28 TCs at any of the FEMA defined communities. Because of the nature of the data (non-negative, discrete and with large percentages of zeros), we use a zero-inflated negative binomial (ZINB) regression model. A ZINB specification allows for over-dispersion resulting from an excessive number of zeroes by splitting the estimation process in two: 1) estimation of a probit model to predict the probability of zero claims within a community; and 2) estimation of a negative binomial (NB) model to predict the number of claims in a given community^5^,^36^. We applied the Vuong test to compare the ZINB and NB models, finding strong statistical evidence in support of the ZINB over the NB.

The predictors are a community’s distance from the coastline (“Miles25–100”, “Miles100–500”, “Miles500+”), whether it is located within a coastal state (“CoastalState”), its percentage of impervious surface (based upon 2006 values; “Impervious”), the proportion of the NFIP community in low, medium or high risk areas (“PropLowRisk”, “PropMedRisk”, “PropHighRisk”), the natural log number of housing units (based on the 2010 values; “LnHousing”), and the natural log of the number of NFIP residential policies-in-force in the community per year of the event (“LnResPol”). The flood ratio is transformed into a dummy variable, with bins of a flood ratio below 1 is for bankfull conditions; a flood ratio between 1 and 1.5 represents minor flooding conditions, while values between 1.5 and 2.2 and larger than 2.2 are indicative of moderate and major flooding, respectively, where bankfull is the omitted dummy variable category. We also control for any unobserved event-specific fixed effects through event dummy variables represented by the TC intensity at landfall – tropical storm, hurricane, and major hurricane with hurricane the omitted category (“TS”, “MajorHurr”). We also include year variables to control for any unobserved time-specific fixed effects with 2001 the omitted category (“YrDummy”). For the inflate portion of the ZINB model, which estimates the probability of zero flood claims occurring in any one community we include the largest flood ratio for a given community (“MaxFR”) and the natural log of the number of NFIP residential policies-in-force in the community per year of the event (“LnResPol”).

Specific data for the non-flood ratio predictors are as follows. The proportion of each community within high, medium and low flood risk according to a 1996 Natural Disaster Study conducted by the Federal Emergency Management Agency on behalf of the U.S. Department of Transportation Pipeline and Hazardous Materials Safety Administration (https://www.npms.phmsa.dot.gov/DisasterData.aspx). The low-, medium- and high-risk areas are derived from a 1-km resolution grid dataset that uses underlying topography and hydrography of the area while estimating the rankings of flood risk on a 0–100 scale that represent relative risk of flood hazard. In the dataset, a scale of 0–69 is indicative of a low risk area, 70–84 is indicative of a medium risk area and 85–100 is indicative a high risk area. For each community we calculated the proportion of area representing three levels of low, medium, and high flood risk. We have also used the numbers of housing units and population obtained from 2010 census blocks (from the 2010 census, U.S. Census Bureau). Distance bands between communities and the coasts are measured from the edges of communities closest to open-ocean coastlines. The information about the percentage of impervious surface is based on the 2006 National Land Cover Database by USGS; the percent change in impervious surfaces was calculated from the 2001 and 2011 datasets. Coastal states include Alabama, Connecticut, Delaware, Florida, Georgia, Louisiana, Maryland, Massachusetts, Mississippi, New Hampshire, New Jersey, New York, North Carolina, Rhode Island, South Carolina, Texas, Virginia, and the District of Columbia. Non-coastal states include Arkansas, Arizona, Colorado, Iowa, Illinois, Indiana, Kansas, Kentucky, Michigan, Minnesota, Missouri, Nebraska, New Mexico, Ohio, Oklahoma, Pennsylvania, Tennessee, Vermont, Wisconsin, and West Virginia.

Lastly, to further assess the accuracy and validity of our estimated ZINB model in Table 1, we perform cross-validation on these models to estimate their ability to fit out-of-sample data. Specifically we perform k-fold cross validation on the models where the model data are randomly split into k partitions. For each k partition, the specified ZINB model is fit (i.e., trained) on k-1 partitions of the data, with the resulting parameters used to predict the dependent variable in the unused group (i.e., the test data). We perform k-fold cross validation using the CROSSFOLD Stata module with k set to both 20 and 30. Thus for k = 20, the sample size is approximately 143,000 (95% of the total 150,000 observations) in the training model estimations, and for k = 30, it is approximately 145,000 (96.7% of the total 150,000 observations). We select these k values as the average number of impacted communities per storm is 5376, so essentially we are trying to predict 1 out of sample TC event. As a measure of the goodness-of-fit from each k-fold validation, we report the generated pseudo-R-squared from CROSSFOLD. The pseudo-R-squared provides the square of the correlation coefficients of the predicted and actual values of the count of the number of flood claims.

## Additional Information

**How to cite this article:** Czajkowski, J. *et al*. Assessing Current and Future Freshwater Flood Risk from North Atlantic Tropical Cyclones via Insurance Claims. *Sci. Rep.* **7**, 41609; doi: 10.1038/srep41609 (2017).

**Publisher's note:** Springer Nature remains neutral with regard to jurisdictional claims in published maps and institutional affiliations.

## Supplementary Material

Supplemental Information

## Figures and Tables

**Table 1:** Statistical modeling of the number of freshwater related residential flood insurance claims using a zero-inflated negative binomial model.

	Estimate	Robust Standard Error	[95% Confidence Intervals]
Minor flooding	0.921***	0.104	[0.718; 1.125]
Moderate flooding	1.579***	0.120	[1.344; 1.814]
Major flooding	2.493***	0.124	[2.250; 2.736]
LnResPol	0.896***	0.044	[0.809; 0.983]
LnHousing	−0.148***	0.042	[−0.231; −0.066]
PropLowRisk	−0.183	0.336	[−0.842; 0.476]
PropMedRisk	0.090	0.340	[−0.576; 0.756]
PropHighRisk	0.623*	0.330	[−0.024; 1.269]
YrDummy2002	−1.079***	0.213	[−1.496; −0.662]
YrDummy2003	−0.329	0.371	[−1.056; 0.398]
YrDummy2004	−4.058***	0.212	[−4.473; −3.643]
YrDummy2005	−3.304***	0.212	[−3.739; −2.870]
YrDummy2006	−2.884***	0.321	[−3.512; −2.260]
YrDummy2007	−2.475***	0.283	[−3.031; −1.920]
YrDummy2008	−1.446***	0.170	[−1.779; −1.113]
YrDummy2011	−1.333***	0.192	[−1.709; −0.958]
YrDummy2012	−0.464**	0.194	[−0.844; −0.085]
TS	−2.400***	0.148	[−2.689; −2.110]
MajorHurr	2.298***	0.113	[2.077; 2.518]
CoastalState	0.029	0.081	[−0.129; 0.187]
Impervious	0.700***	0.182	[0.344; 1.055]
Miles25-100	−0.561***	0.095	[−0.748; −0.375]
Miles100-500	−0.593***	0.103	[−0.796; −0.390]
Miles500 +	−1.422***	0.367	[−2.146; −0.697]
Intercept	−1.248***	0.398	[−2.028; −0.468]
Inflate			
MaxFR	−1.436***	0.032	[−1.498; −1.373]
LnResPol	−0.120***	0.010	[−0.139; −0.010]
Intercept	2.427***	0.063	[2.303; 2.549]
Ln(Dispersion α)	1.517***	0.041	[−1.437; −1.596]
Dispersion α	4.555***	0.185	[4.207; 4.932]

There are 150,546 total observations, of which 6,631 are non-zero. The predictors are a community’s distance from the coastline (“Miles25–100”, “Miles100–500”, “Miles500+”, where miles 0 to 25 is the omitted dummy variable category), whether it is located within a coastal state (“CoastalState”), its percentage of impervious surface (based upon 2006 values; “Impervious”), the proportion of the NFIP community in low, medium or high risk areas (“PropLowRisk”, “PropMedRisk”, “PropHighRisk”), the natural log of the number of housing units (based on the 2010 values; “LnHousing”), and the natural log of the number of NFIP residential policies-in-force in the community per year of the event (“LnResPol”). The flood ratio is transformed into a dummy variable, with bins of a flood ratio below 1 is for bankfull conditions; a flood ratio between 1 and 1.5 represents minor flooding conditions, while values between 1.5 and 2.2 and larger than 2.2 are indicative of moderate and major flooding, respectively, where bankfull is the omitted dummy variable category. We also control for any unobserved event-specific fixed effects through event dummy variables represented by the TC intensity at landfall – tropical storm, hurricane, and major hurricane with hurricane the omitted category (“TS”, “MajorHurr”). We also include year variables to control for any unobserved time-specific fixed effects with 2001 the omitted category (“YrDummy”). The zero-inflated part of the model uses as predictors the largest flood ratio for a given community (“MaxFR”) and the natural log of the number of NFIP residential policies-in-force in the community per year of the event (“LnResPol”). The results for the coefficient of dispersion α are also reported, highlighting that the data are overdispersed (α larger than 0). The symbols “***”, “**”, “*” refer to coefficients that are different from zero at the 0.01, 0.05, and 0.10 significance level, respectively. Consult the Methods section for more details on the statistical estimation employed.

**Table 2:** Percentage increase in the number of flood claims resulting from 1%, 5%, 10%, and 20% increases in the values of the flood ratio, and from increasing urbanization.

	Percentage increase in the number of flood claims
1% increase in flood ratio	0.7%
5% increase in flood ratio	3.5%
10% increase in flood ratio	8.6%
20% increase in flood ratio	17.1%
Urbanization increase	2.4%
