# Supplementary material for: Assessing Current and Future Freshwater Flood Risk from North Atlantic Tropical Cyclones via Insurance Claims
Source: Sci Rep. 2017 Feb 2;7:41609. doi: 10.1038/srep41609 (PMC5288645; doi:10.1038/srep41609)
Supplement: Supplemental Information [file srep41609-s1.pdf]

# Assessing Current and Future Freshwater Flood Risk from North Atlantic Tropical Cyclones via Insurance Claims

**Associated Supplemental Material –** December 6, 2016

JEFFREY CZAJKOWSKI[1,2], GABRIELE VILLARINI[3], MARILYN MONTGOMERY[1], ERWANN MICHEL-KERJAN[1] AND RADOSLAW GOSKA[3]

[1]The Wharton School, University of Pennsylvania, 3730 Walnut Street, Philadelphia, PA 19104, USA

[2]Willis Research Network, 51 Lime Street, London EC3M 7DQ, UK

[3]IIHR-Hydroscience & Engineering, The University of Iowa, 100 C Maxwell3 Stanley Hydraulics Laboratory, Iowa City, IA 52242, USA

Corresponding author: Jeffrey Czajkowski, The Wharton School, University of Pennsylvania, Huntsman Hall, Suite 500, 3730 Walnut Street, Philadelphia, PA, 19104, USA; jczaj@wharton.upenn.edu; +(1) 215 898-8047

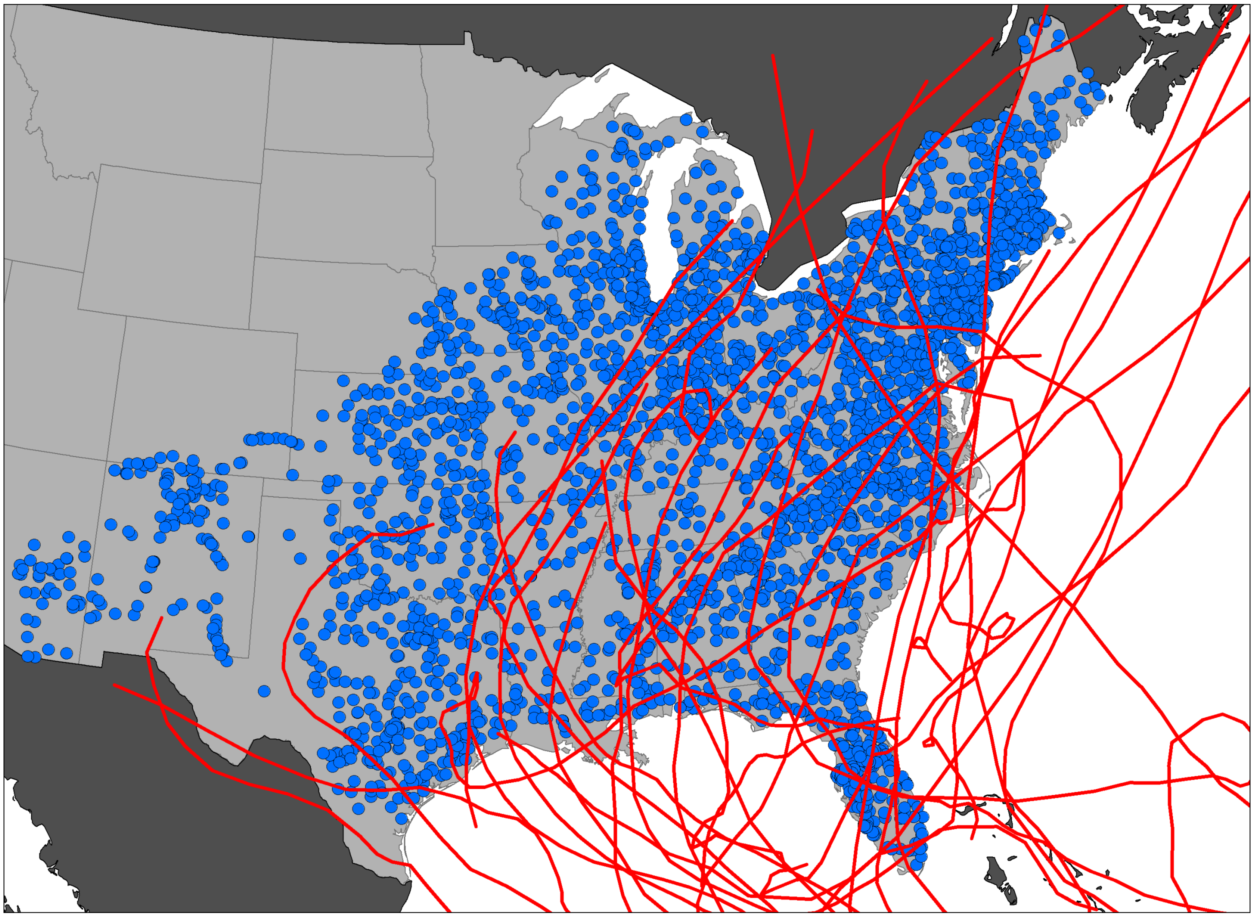

SUPPLEMENTARY FIGURE 1. Map showing the location of the 3035 U.S. Geological Survey (USGS) stream gages (blue circles) within 500 km of at least one the 28 tropical cyclones (TCs; red lines) considered in this study. Maps were created in ESRI ArcGIS version 10.2.2 (http://www.esri.com/software/arcgis/arcgis-for-desktop).

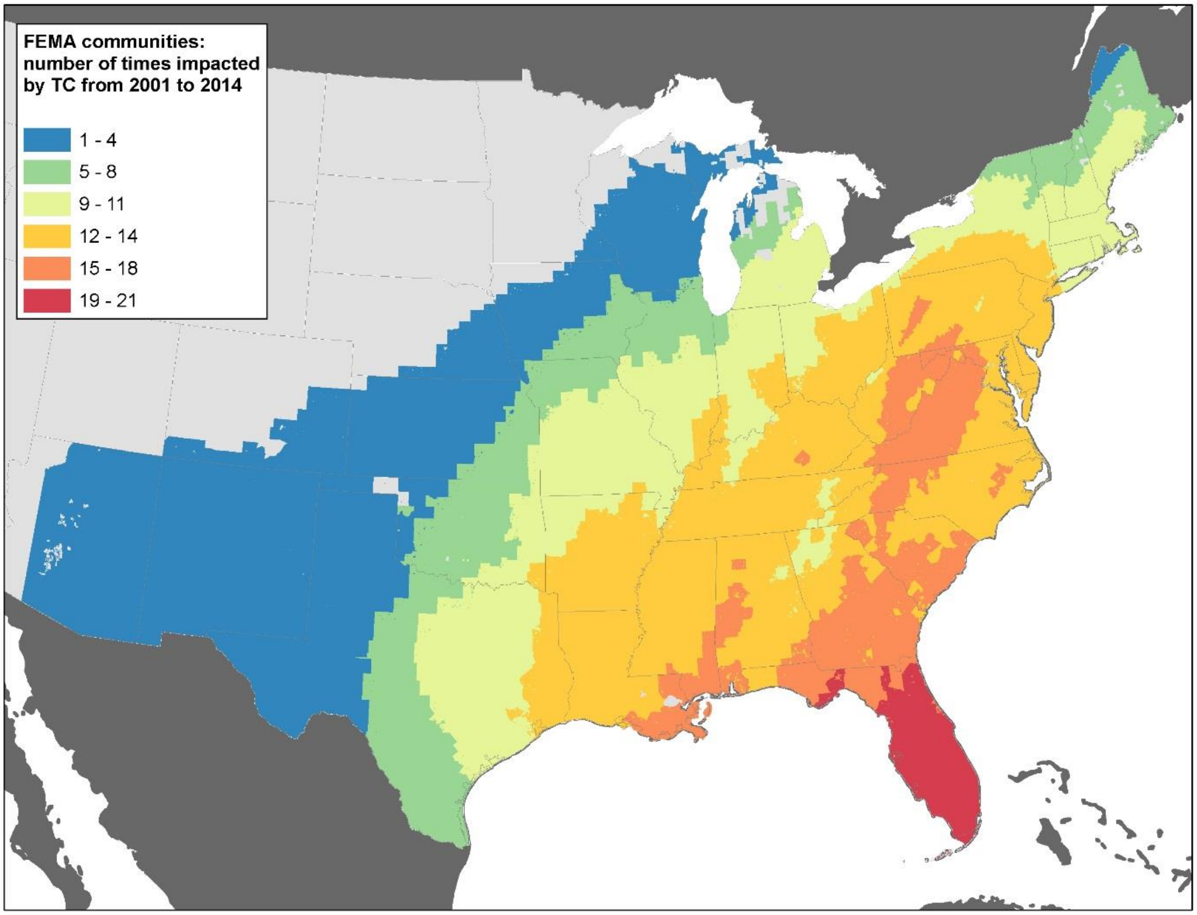


SUPPLEMENTARY FIGURE 2. FEMA's NFIP communities affected by TCs. This figure shows the number of times any one FEMA defined community was affected by any of the 28 TCs considered in this study. Maps were created in ESRI ArcGIS version 10.2.2 (http://www.esri.com/software/arcgis/arcgis-for-desktop).

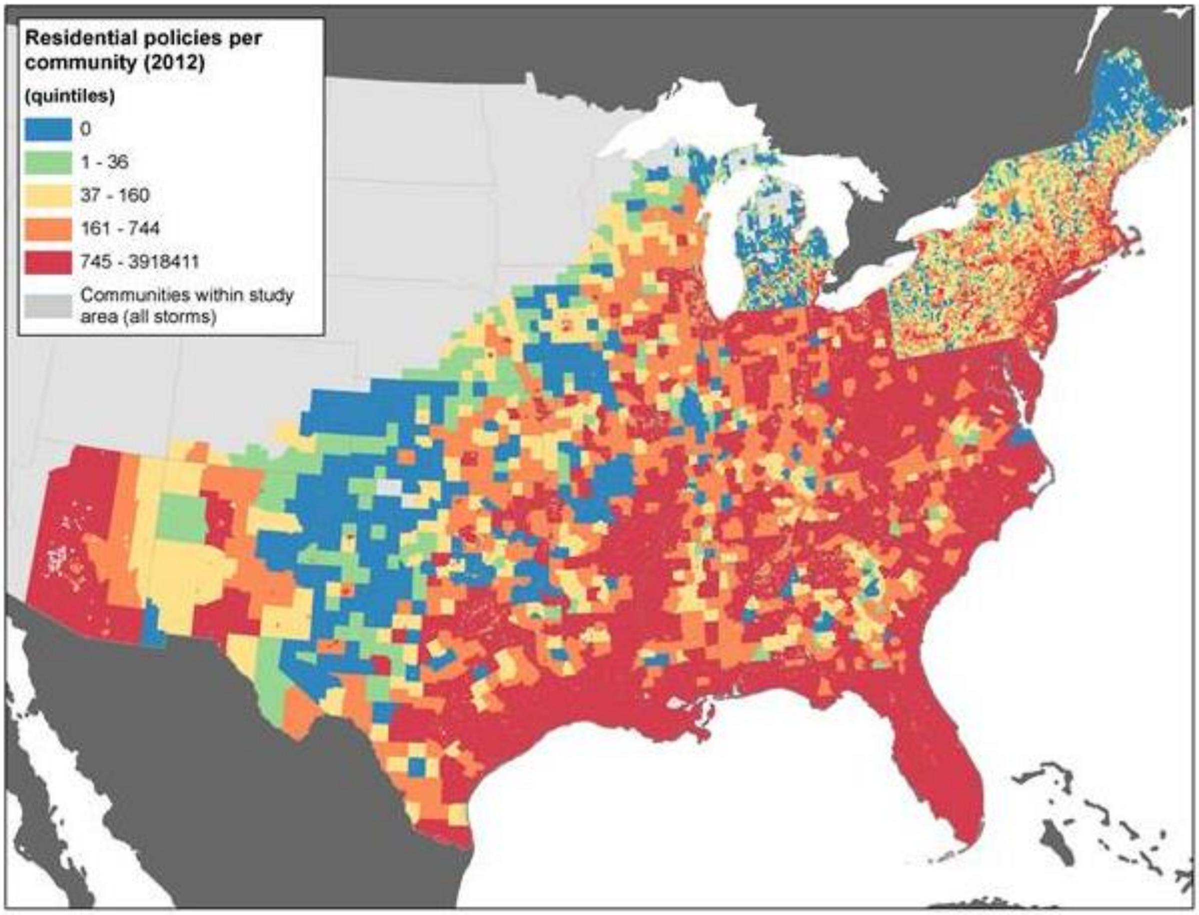


SUPPLEMENTARY FIGURE 3. NFIP total residential flood insurance policies in-force per community as per 2012. Maps were created in ESRI ArcGIS version 10.2.2 (http://www.esri.com/software/arcgis/arcgis-for-desktop).

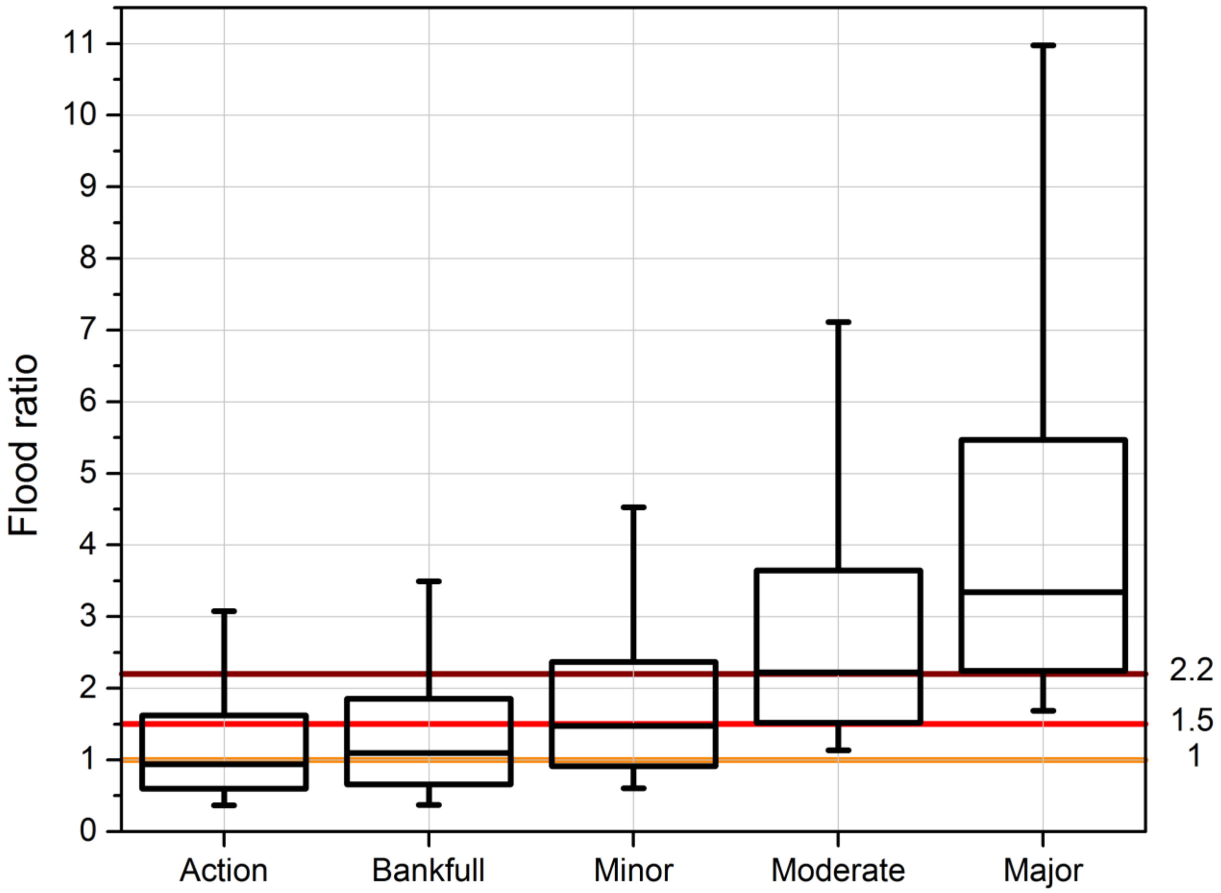


SUPPLEMENTARY FIGURE 4. Relating the flood ratio to flood status classification. This figure shows the relationship between flood ratio values and flood status classification according to the National Weather Service (Action, Bankfull, Minor, Moderate and Major Flooding) for all the stations in our domain. In each boxplot, the limits of the whiskers represent the 0.1-0.9 quantiles, the limits of the box the 0.25 and 0.75 quantiles, while the line in the middle of the box the median (0.5 quantile). The colored horizontal lines represent the limits of the three categories in which we have stratified the flood ratios: a flood ratio below 1 is for bankfull conditions; a flood ratio between 1 and 1.5 represents minor flooding conditions, while values between 1.5 and 2.2 and larger than 2.2 are indicative of moderate and major flooding, respectively.

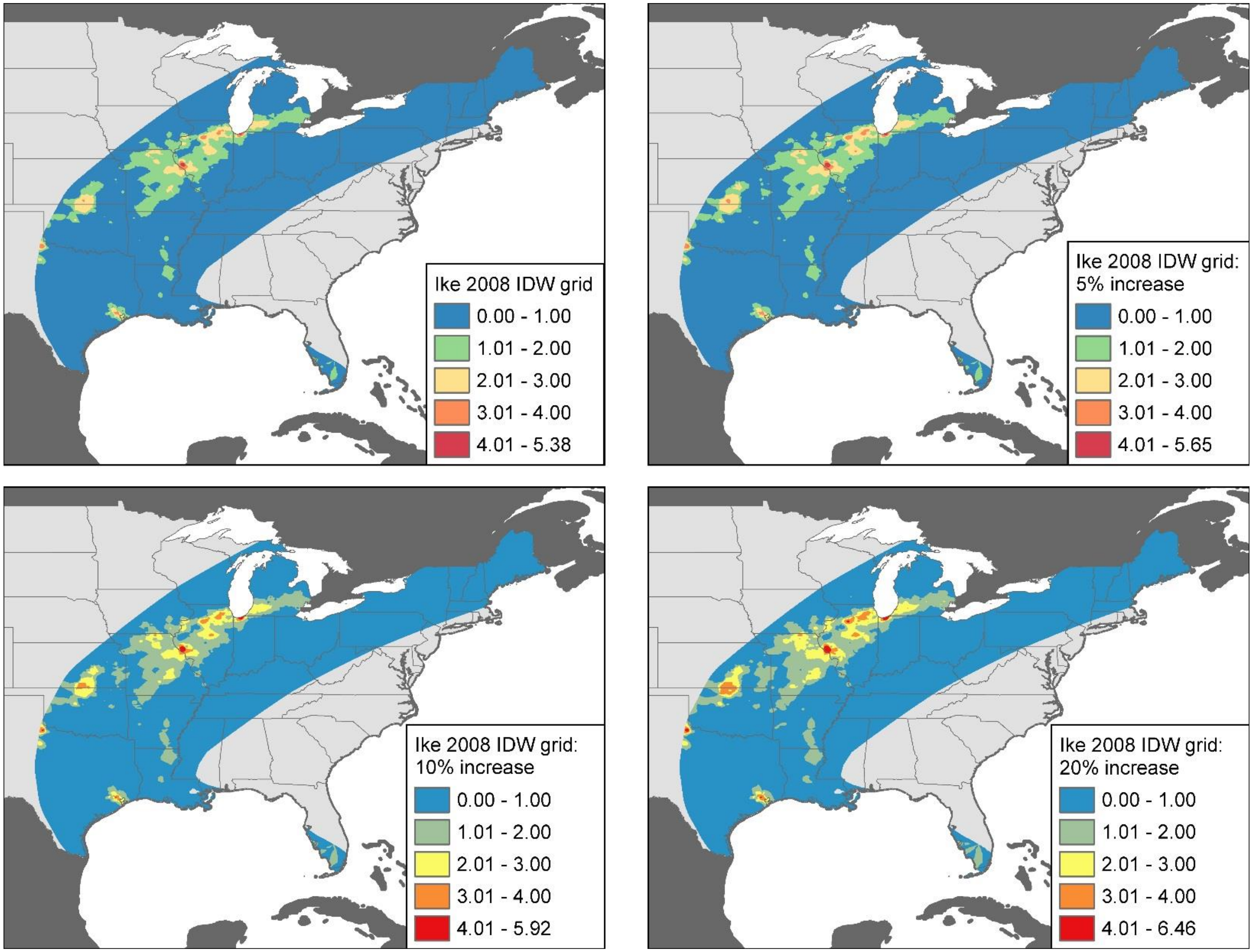


SUPPLEMENTARY FIGURE 5. Increasing the observed Hurricane Ike TC flood ratios by 1, 5, 10, and 20 percent at every USGS location. Maps were created in ESRI ArcGIS version 10.2.2 (http://www.esri.com/software/arcgis/arcgis-for-desktop).

SUPPLEMENTARY TABLE 1. Summary of the 28 TCs considered in this study. The information aboutthe radius at landfall is based on the Extended Best Track data.

| # | Year | Storm | Wind speed at landfall (knots) | Category | Radius at landfall (nm) |
|---|---|---|---|---|---|
| 1 | 2001 | ALLISON | 45 | TS | 120 |
| 2 | 2001 | BARRY | 60 | TS | 175 |
| 3 | 2001 | GABRIELLE | 60 | TS | 180 |
| 4 | 2002 | ISIDORE | 55 | TS | 360 |
| 5 | 2002 | LILI | 80 | CAT1 | 200 |
| 6 | 2003 | CLAUDETTE | 80 | CAT1 | 180 |
| 7 | 2003 | ISABEL | 90 | CAT2 | 300 |
| 8 | 2004 | ALEX* | 85 | CAT2 | 150 |
| 9 | 2004 | CHARLEY | 130 | CAT4 | 100 |
| 10 | 2004 | FRANCES | 90 | CAT2 | 200 |
| 11 | 2004 | IVAN | 105 | CAT3 | 240 |
| 12 | 2004 | JEANNE | 105 | CAT3 | 200 |
| 13 | 2005 | DENNIS | 105 | CAT3 | 300 |
| 14 | 2005 | KATRINA | 110 | CAT3 | 325# |
| 15 | 2005 | OPHELIA** | 75 | CAT1 | 200 |
| 16 | 2005 | RITA | 100 | CAT3 | 300 |
| 17 | 2005 | WILMA | 105 | CAT3 | 300 |
| 18 | 2006 | ALBERTO | 40 | TS | 175 |
| 19 | 2006 | ERNESTO | 40 | TS | 125 |
| 20 | 2007 | ERIN | 30 | TD | 150 |
| 21 | 2008 | DOLLY | 75 | CAT1 | 180 |
| 22 | 2008 | FAY | 55 | TS | 200 |
| 23 | 2008 | GUSTAV | 90 | CAT2 | 232.5# |
| 24 | 2008 | IKE | 95 | CAT2 | 325 |
| 25 | 2011 | IRENE | 75 | CAT1 | 400 |
| 26 | 2012 | DEBBY | 65 | CAT1 | 300 |
| 27 | 2012 | ISAAC | 70 | CAT1 | 275 |
| 28 | 2012 | SANDY | 70 | CAT1 | 512.5# |

* For Hurricane Alex we used the location off of the Carolinas Coast (2004/08/03, 1200) as the closest landfall point.

**For Hurricane Ophelia we used the location off of the Carolinas Coast (2005/09/14, 1800) rather than the landfall location in Florida

# Average value between two time steps.

SUPPLEMENTARY TABLE 2A. Statistical modeling of the number of SFHA freshwater flood insurance claims (329,301total claims) using a zero-inflated negative binomial model. (SFHA: special flood hazard areas, defined by FEMA as high risk zones). Predictors are same as those in Table 1.

| | Estimate | Robust Standard Error | [95% Confidence Intervals] |
|---|---|---|---|
| Minor flooding | 0.950*** | 0.121 | [0.713; 1.188] |
| Moderate flooding | 1.478*** | 0.136 | [1.212; 1.745] |
| Major flooding | 2.322*** | 0.133 | [2.062; 2.582] |
| LnResPol | 0.920*** | 0.044 | [0.833; 1.007] |
| LnHousing | -0.183*** | 0.041 | [-0.265; -0.102] |
| PropLowRisk | -0.217 | 0.348 | [-0.9; 0.465] |
| PropMedRisk | 0.302 | 0.354 | [-0.392; 0.997] |
| PropHighRisk | 0.687** | 0.342 | [0.017; 1.358] |
| YrDummy2002 | -0.654*** | 0.241 | [-1.128; -0.182] |
| YrDummy2003 | 0.418 | 0.476 | [-0.515; 1.352] |
| YrDummy2004 | -3.851*** | 0.227 | [-4.296; -3.406] |
| YrDummy2005 | -3.035*** | 0.240 | [-3.506; -2.564] |
| YrDummy2006 | -2.735*** | 0.422 | [-3.562; -1.909] |
| YrDummy2007 | -3.158*** | 0.375 | [-3.893; -2.423] |
| YrDummy2008 | -1.454*** | 0.171 | [-1.791; -1.118] |
| YrDummy2011 | -1.181*** | 0.202 | [-1.577; -0.786] |
| YrDummy2012 | -0.037 | 0.212 | [-0.452; 0.378] |
| TS | -2.529*** | 0.147 | [-2.818; -2.241] |
| MajorHurr | 2.164*** | 0.129 | [1.911; 2.417] |
| CoastalState | -0.249*** | 0.089 | [-0.424; -0.076] |
| Impervious | 0.500** | 0.207 | [0.094; 0.906] |
| Miles25-100 | -0.596*** | 0.102 | [-0.796; -0.397] |
| Miles100-500 | -0.523*** | 0.110 | [-0.738; -0.309] |
| Miles500+ | -1.304*** | 0.423 | [-2.133; -0.476] |
| Intercept | -1.295*** | 0.415 | [-2.109; -0.481] |

| Inflate | Estimate | Robust Standard Error | [95% Confidence Intervals] |
|---|---|---|---|
| MaxFR | -1.375*** | 0.033 | [-1.439; -1.311] |
| LnResPol | -0.168*** | 0.011 | [-0.191; -0.146] |
| Intercept | 2.780*** | 0.071 | [2.641; 2.919] |

| | Estimate | Robust Standard Error | [95% Confidence Intervals] |
|---|---|---|---|
| Ln(Dispersion $\alpha$) | 1.661*** | 0.041 | [1.58; 1.742] |
| Dispersion $\alpha$ | 5.264*** | 0.218 | [4.854; 5.709] |

SUPPLEMENTARY TABLE 2B. Statistical modeling of the number of Non-SFHA freshwater flood insurance claims (114,183 total claims) using a zero-inflated negative binomial model. (SFHA: special flood hazard areas, defined by FEMA as high risk zones). Predictors are same as those in Table 1.

| | Estimate | Robust Standard Error | [95% Confidence Intervals] |
|---|---|---|---|
| Minor flooding | 0.513*** | 0.114 | [0.29; 0.736] |
| Moderate flooding | 1.271*** | 0.125 | [1.026; 1.517] |
| Major flooding | 1.913*** | 0.127 | [1.664; 2.162] |
| LnResPol | 0.787*** | 0.041 | [0.707; 0.868] |
| LnHousing | -0.047 | 0.038 | [-0.122; 0.027] |
| PropLowRisk | -0.242 | 0.654 | [-1.525; 1.039] |
| PropMedRisk | -0.229 | 0.658 | [-1.519; 1.061] |
| PropHighRisk | 0.484 | 0.655 | [-0.8; 1.769] |
| YrDummy2002 | -1.472*** | 0.195 | [-1.855; -1.089] |
| YrDummy2003 | -1.497*** | 0.256 | [-2; -0.995] |
| YrDummy2004 | -3.738*** | 0.212 | [-4.153; -3.322] |
| YrDummy2005 | -3.047*** | 0.246 | [-3.528; -2.565] |
| YrDummy2006 | -2.673*** | 0.437 | [-3.529; -1.817] |
| YrDummy2007 | -1.877*** | 0.301 | [-2.467; -1.287] |
| YrDummy2008 | -1.182*** | 0.170 | [-1.516; -0.849] |
| YrDummy2011 | -1.242*** | 0.195 | [-1.625; -0.859] |
| YrDummy2012 | -0.815*** | 0.197 | [-1.203; -0.429] |
| TS | -1.878*** | 0.158 | [-2.187; -1.568] |
| MajorHurr | 2.086*** | 0.119 | [1.853; 2.32] |
| CoastalState | 0.244*** | 0.076 | [0.095; 0.393] |
| Impervious | 0.881*** | 0.186 | [0.518; 1.246] |
| Miles25-100 | -0.330*** | 0.078 | [-0.484; -0.178] |
| Miles100-500 | -0.526*** | 0.095 | [-0.714; -0.34] |
| Miles500+ | -1.788*** | 0.407 | [-2.586; -0.99] |
| Intercept | -2.473*** | 0.702 | [-3.849; -1.096] |

| Inflate | Estimate | Robust Standard Error | [95% Confidence Intervals] |
|---|---|---|---|
| MaxFR | -1.300*** | 0.036 | [-1.371; -1.229] |
| LnResPol | -0.066*** | 0.010 | [-0.087; -0.046] |
| Intercept | 2.215*** | 0.080 | [2.059; 2.372] |

| | Estimate | Robust Standard Error | [95% Confidence Intervals] |
|---|---|---|---|
| Ln(Dispersion $\alpha$) | 1.366*** | 0.042 | [1.284; 1.447] |
| Dispersion $\alpha$ | 3.918*** | 0.163 | [3.612; 4.251] |

SUPPLEMENTARY TABLE 2C. Statistical modeling of the number of freshwater flood insurance claims for Single-Family residential properties only (374,306 total claims) using a zero-inflated negative binomial model. Predictors are same as those in Table 1.

| | Estimate | Robust Standard Error | [95% Confidence Intervals] |
|---|---|---|---|
| Minor flooding | 0.886*** | 0.103 | [0.685; 1.088] |
| Moderate flooding | 1.545*** | 0.118 | [1.313; 1.777] |
| Major flooding | 2.473*** | 0.123 | [2.231; 2.714] |
| LnResPol | 0.885*** | 0.046 | [0.796; 0.975] |
| LnHousing | -0.145*** | 0.043 | [-0.23; -0.062] |
| PropLowRisk | -0.239 | 0.353 | [-0.931; 0.453] |
| PropMedRisk | 0.035 | 0.357 | [-0.664; 0.736] |
| PropHighRisk | 0.530 | 0.348 | [-0.151; 1.212] |
| YrDummy2002 | -1.138*** | 0.195 | [-1.52; -0.756] |
| YrDummy2003 | -0.388 | 0.365 | [-1.103; 0.326] |
| YrDummy2004 | -4.090*** | 0.210 | [-4.502; -3.679] |
| YrDummy2005 | -3.363*** | 0.219 | [-3.792; -2.934] |
| YrDummy2006 | -2.854*** | 0.316 | [-3.473; -2.235] |
| YrDummy2007 | -2.415*** | 0.302 | [-3.007; -1.823] |
| YrDummy2008 | -1.426*** | 0.169 | [-1.757; -1.095] |
| YrDummy2011 | -1.391*** | 0.190 | [-1.762; -1.019] |
| YrDummy2012 | -.4757** | 0.193 | [-0.855; -0.097] |
| TS | -2.409*** | 0.146 | [-2.696; -2.123] |
| MajorHurr | 2.306*** | 0.114 | [2.083; 2.529] |
| CoastalState | 0.018 | 0.081 | [-0.14; 0.178] |
| Impervious | 0.369** | 0.182 | [0.012; 0.726] |
| Miles25-100 | -0.569*** | 0.096 | [-0.758; -0.381] |
| Miles100-500 | -0.6436*** | 0.102 | [-0.844; -0.444] |
| Miles500+ | -1.384*** | 0.374 | [-2.116; -0.651] |
| Intercept | -1.144*** | 0.414 | [-1.954; -0.333] |

| Inflate | Estimate | Robust Standard Error | [95% Confidence Intervals] |
|---|---|---|---|
| MaxFR | -1.437*** | 0.032 | [-1.501; -1.373] |
| LnResPol | -0.119*** | 0.010 | [-0.140; -0.100] |
| Intercept | 2.432*** | 0.065 | [2.304; 2.559] |

| | Estimate | Robust Standard Error | [95% Confidence Intervals] |
|---|---|---|---|
| Ln(Dispersion $\alpha$) | 1.515*** | 0.041 | [1.434; 1.597] |
| Dispersion $\alpha$ | 4.551*** | 0.189 | [4.196; 4.936] |

SUPPLEMENTARY TABLE 3. Cross Validation Goodness-of-Fit Pseudo R Squared Statistics

| (k-1) Training Estimate | *k=20* | *k=30* |
|---|---|---|
| est1 | 0.04 | 0.01 |
| est2 | 0.10 | 0.01 |
| est3 | 0.15 | 0.01 |
| est4 | 0.07 | 0.09 |
| est5 | 0.06 | 0.03 |
| est6 | 0.10 | 0.02 |
| est7 | 0.02 | 0.07 |
| est8 | 0.09 | 0.39 |
| est9 | 0.02 | 0.03 |
| est10 | 0.06 | 0.04 |
| est11 | 0.20 | 0.30 |
| est12 | 0.13 | 0.26 |
| est13 | 0.01 | 0.10 |
| est14 | 0.04 | 0.34 |
| est15 | 0.00 | 0.14 |
| est16 | 0.01 | 0.02 |
| est17 | 0.02 | 0.52 |
| est18 | 0.02 | 0.01 |
| est19 | 0.23 | 0.02 |
| est20 | 0.02 | 0.15 |
| est21 | | 0.43 |
| est22 | | 0.06 |
| est23 | | 0.01 |
| est24 | | 0.45 |
| est25 | | 0.05 |
| est26 | | 0.04 |
| est27 | | 0.02 |
| est28 | | 0.03 |
| est29 | | 0.05 |
| est30 | | 0.02 |
| **mean pseudo-R2** | **0.07** | **0.13** |
